# Supplementary material for: Accessing medicines for non-communicable diseases: Patients and health care workers’ experiences at public and private health facilities in Uganda
Source: PLoS One. 2020 Jul 7;15(7):e0235696. doi: 10.1371/journal.pone.0235696 (PMC7340292; doi:10.1371/journal.pone.0235696)
Supplement: S1 File — (DOCX) [file pone.0235696.s001.docx]

**Qualitative guides**

1. **Key informant interview guide**

This interview guide will be used to collect information from health facility managers and heads of the pharmacy department.

**Identification of key informant**

District: ……………………………..

Position: ………………………………

Health facility name: ……………………………

**Questions**

1. As a health practitioner at this facility, can you tell me about yourself?
2. What are your responsibilities in this facility/ what do you do? (Probe for; actual position/ delegated tasks, work experience especially in handling NCDs, how long they’ve worked in the facility).
3. What are the common NCDs that are managed in this facility? (*You may have to explain meaning of NCDs*) (probe for Diabetes and hypertension if not mentioned)
4. Let’s now take a moment and talk more about management of diabetes and hypertension (probe: care at facility, how patients are attended to, and clinic days, handling emergency cases).

(*Get to know how the DM/ Hyp clinic runs in the facility, is there a special class on insurance? How are services provided in this clinic?)*

1. Let’s talk about essential medicines for diabetes and hypertension. (Probe for: the procurement process, available drugs, prescription and dispensing procedure)
2. What are some of the challenges you face in the management of diabetes mellitus and hypertension at this facility? (Probe if not mentioned; cost, volume of patients, stock outs).
3. In your opinion, what could be hindering the patients specifically those suffering from diabetes and hypertension from getting the prescribed medicines? (Probe. Cost, Insufficient drugs, )
4. What happens when a patient is not able to get the prescribed medicines at the facility? (Probe for alternatives besides buying at pharmacies eg, use of herbs, visit other facilities/ multiple consultations, nothing done!).
5. **Patient in-depth guide**

District: ……………………………………..

Health facility name: ……………………..

Age ……………………………………..

Gender: ………………………………..

**Questions**

1. As person who has come to attend the DM/HTN clinic today, can you tell me you got to know that you were hypertensive or diabetic. (Probe: when, what did you experience/ signs before you were diagnosed / confirmed?)
2. what did you do when you realized that you have this condition?
3. What influenced you to come to this facility to seek for medication? (Probe: place of residence, distance moved and transport costs).
4. Can you comment about the treatment you receive for this condition (probe for: availability of specialist for consultations, working hours, waiting time, availability of medicines for the condition) (*ask for the time patient came and time patient was attended to*).
5. Let’s now talk about the process of getting the medicines prescribed for you today.
6. Briefly talk about the process, where did you start from, talk about the prescribing and dispensing process that you have gone through up to the time when you left the pharmacy.
7. Which drugs have been prescribed for you? Were you able to get them from here today?
8. Could you please give reasons for getting or not getting the medicines (probe for waiting time, cost, and stock out)

(Probe for m*edicines which are never at the facility but always prescribed for you to buy from other pharmacies*)

1. Have you had any side effects of drugs that you receive and how do they affect you? What do you do after getting these side effects?
2. If you are not provided with the medicines prescribed to you, what do you do? (Probe. alternative drugs/ medication eg. Herbal medication, visit other facilities )
3. What do you think can be done for you to get all your medicines from this health facility?
4. **Focus group discussion**

District: ……………………………………..

Health facility name: ……………………..

Each participant: age, gender……….

**Questions**

1. As persons who have come to attend the DM/HTN clinic today, could you talk about the health conditions you have. (How you got to know it, when, what did you experience/ signs before you were diagnosed / confirmed?
2. Since you were diagnosed with Diabetes mellitus or hypertension, what have you been doing to manage your health condition (Probe; health seeking behavior, heath facilities visited )
3. What are some of the challenges you encounter in the management of your health condition?
4. Let’s now talk about the treatment you receive for these conditions? (Probe for: availability of specialist for consultations, working hours, waiting time, availability of medicines for the condition) (*ask for the time patients came and when attended to*).
5. Let’s now talk about the process of getting the medicines prescribed for you today.
6. Briefly talk about the process you go through from arrival. (Talk about the prescribing and dispensing process that they have gone through up to the time when each left the pharmacy).
7. Which drugs have been prescribed for you today? Did you get them?
8. Could you please give reasons for getting or not getting the medicines (probe for waiting time, cost, and stock out). (*Probe for medicines which are never at the facility but always prescribed for you to buy from other pharmacies*).
9. Have you had any side effects of drugs that you receive and how do they affect you? What do you do after getting these side effects?
10. If you are not provided with the medicines prescribed to you, what do you do? (Probe. alternative drugs/ medication eg. Herbal medication, prayers)
11. Do you make consultations elsewhere or at other facilities?
12. What do you think can be done for you to get all your medicines from this health facility?
