# Supplementary material for: Accessing medicines for non-communicable diseases: Patients and health care workers’ experiences at public and private health facilities in Uganda
Source: PLoS One. 2020 Jul 7;15(7):e0235696. doi: 10.1371/journal.pone.0235696 (PMC7340292; doi:10.1371/journal.pone.0235696)
Supplement: Attachment — Submitted filename: Response to Reviewers.docx [file pone.0235696.s003.docx]

5^th^ May, 2020

**Accessing Medicines for Non-Communicable Diseases: Patients and Health Care Workers’ Experiences at Public and Private Health Facilities in Uganda**

**(PONE-D-20-02052)**

Dear Editor,

The comments are addressed below, and corresponding changes in the revised manuscript with track changes.

**Additional Editor Comment**:

This is an important study addressing the issue related with access to essential medicines for NCDs covering both public and private sectors. To strengthen scientific integrity and applicability of research evidence for Universal Health Coverage, authors have to revise extensively. Moreover, English language correction is deemed necessary.

**Response:** Thank you for the comment. We have reviewed the manuscript and made corrections to the language errors.

**Reviewers' comments:**

**Reviewer #1:**

Thank you for giving me the opportunity to review this manuscript.

The manuscript is well written and addresses an important public health problem. However, the manuscript requires major revision in terms of methods and results section.

**General comments:** Entire manuscript requires proofreading for typographical and grammatical errors.

**Response:** We have proof read the entire manuscript and made corrections to typographical and grammatical errors.

**Introduction:
Comment:** Background and rationale are well written. However, further points on the necessity and application of current study findings is required.
**Response:** Thank you for this comment. We have added more information that that highlights the necessity and application of our study findings (Background; Pages 3-4, Lines 80 -84).

**Methods:**

**Comment:** Study setting: Details on distribution of NCD patients receiving care at public sector and private sector can be provided. Public and private sector distribution can be provided.

**Response:** We have included the distribution of public and private sector facilities in the country. We could not provide the distribution of NCD patients receiving care at public and private sector since presently, to the best of our knowledge, there is no documentation about this in Uganda. We however, have stated the high concentration of NCD service provision at public sector hospitals and hence high NCD patient volumes at these facilities. (Methods; Pages 4-5, Lines 100 - 108).

**Comment:** Study design: Can be mentioned as qualitative descriptive instead of cross-sectional qualitative
**Response:** This statement has been rephrased to mention **“**Qualitative descriptive” (Methods; Page 5, Line 111). This change has also been applied to the abstract (Page 2, Line 41).

**Comment:** Sampling strategy: total number of facilities in this region can be provided to see how much the sample is representative. Case load of this hospitals can also be provided. Maximum variation sampling can be mentioned here as it comes up only in overcoming the limitation part.
**Response:** The total number of facilities we selected from has been provided and the number of private not for profit facilities under the IMC network also stated (Methods; Page 5, Lines133 - 144).

We are not able to state the case load for the hospitals since currently there is no documentation on NCD case load of these facilities. However, as previously noted, provision of NCD services is highly concentrated at public hospitals, which have high NCD patient volumes.

A statement about maximum variation sampling has been added (methods; Page 7, line 149).

**Comment:** Table 1: Why there is over-representation of public health facilities and only 2 FGD done in private health facilities.
**Response:** Private for profit public facilities (IMC) and Kabarole hospital had a very low turn-up of patients, which could not support mobilization of participants for FGD. We were able to conduct the two FGD at Lubaga hospital, a private not for profit facility, which had a substantially high patient volume. This detail has been included in the manuscript body (Methods; Page 7, Lines 162 - 164).

**Comment:** Data collection and management: Details on why these many number of FGD, IDI and KII was done can be mentioned. Was it done till data saturation or pre-fixed numbers?
**Response:** We have mentioned the reason for the many FGD, IDI and KII (Methods; Page 7, lines 159 - 160).

**Comment:** Table 3: Framework can be split into two: As patient perspective and health provider perspectives as it will help in exploring both their perspectives. Barriers and facilitators is also not differentiated and mixed up in the same framework. Hence, under each theme, barriers and facilitators can be stated in bracket.
**Response:** Thank you for this guidance. We were not able to split the framework into two since most of the patient and provider perspectives are cross cutting under each theme. We have, however, inserted symbols to denote the perspectives of patients, perspectives of providers and the perspectives of both patients and providers. We have also differentiated barriers and facilitators by stating it in brackets (Results; Pages 10-11, Lines 227 - 228).

**Discussion**

**Comment:** Discussion is very well written by summarizing the main study findings and comparing and contrasting with previous studies with relevant recommendation for public health action and future studies. Limitations in the study is also provided. However, a small suggestion is that the author can consider removing the second paragraph in discussion can be removed as it anyways gets repeated throughout the discussion part.
**Response:** We have deleted the second paragraph (Discussion; Page 20, Lines 446 - 453).

**Reviewer #2:**

This is an important study in the under-examined field of medication access in Uganda. As chronic diseases take over the disease burden in this country and the surrounding region - concurrent with a rise in private-sector care - examination of barriers and facilitators to obtaining medications for non-communicable disease in private and public facilities becomes ever more urgent. I feel the study is robust and its conclusions rigorous and easy to follow. My only comments - for a suggested minor revision - relate to clarity of writing and some remaining questions regarding the methods and implications of the work. Details by manuscript line are as below:

**Comment: Abstract**: would remove "interestingly" from the results section (line 55) and expound a bit more on actionable next steps in the conclusion section (lines 59-60) - for instance why these three proposed solutions are most likely to succeed across both public and private sectors. Would add commas to line 51.
**Response:** The word interestingly has been deleted (Page 2, Line 55), and a reason why the three proposed solutions are likely to succeed both public and private sectors has been added (Page 3, line 59 ). Commas have been added to statement in line 51.

**Comment: Methods:** would change to "works on a referral basis" in line 87. Also kindly clarify the "apparently influence of diabetes patient groups" in lines 109 - 111: who are these groups; how do they influence medication access in Uganda; and what did you feel that their chairpersons would add to the study if interviewed? Lines 129-130 allude to this issue but the specifics remain unclear. How did you select the hospitals and clinics (line 114) apart from geographic diversity? Also, how did you decide (lines 126-127) which patients were assigned to an FGD or IDI? Did patients get to select one of these options, or did you do so? Lastly, please clarify the Framework Method further in lines 156-159 - how does it differ from other analytic strategies in which themes should be analyzed concurrent with contextual review?

**Response:** The statement has been rephrased to read “works on a referral basis” (Page 4, line 92).

We have added more information describing the patient groups and the important role of the chairpersons (Page 5, lines 124 -129).

We purposively selected the study sites (facilities) following geographic diversity. This detail has been stated in the methods section (Page 6, lines 133 -144).

We did not have a criterion for which patient joins an FGD or IDI. We first held IDI and then later held FGD at each facility. It was us (research assistant) who would invite a patient to participate in what was being conducted at that time, either to be interviewed or to be part of an FGD. This detail has been added to the data collection section (Page 8, lines 179 -182).

Details about how analysis is done in the Framework Method is highlighted (Page 8, lines 192 - 196).

**Comment: Results:** generally clear and straightforward, in my view. I would change some passive-voice sentences (lines 246, 339) to active for readability.
**Response:** Thank you, these statements have been rephrased as suggested (Page 14, lines 286; & Page 18, line 380) . The statements now read starting, “We found that ...”.

**Comment:** Discussion: the first few summary lines (400-404) are somewhat redundant and could be shortened or omitted. Similarly, the paragraph from 405-412 - which largely states results rather than analyzing them - could be deleted or moved to the results section. More generally, I'd be excited to see the discussion focus more on potential solutions than the underlying cause of the problems with access. What, for instance, is the PATH Coalition doing now, and what might it do better going forward, in promoting NCD medication access in Uganda? The final paragraph from lines 517 to 532 discusses such steps, but would benefit from more detail if possible - for instance around the nature of innovative patient-centered delivery platforms as mentioned in lines 496-499. Lastly, please note typos in lines 430 and 439.

**Response:** We have deleted the redundant statements (page 20, lines 443-453).

More information of what PATH Coalition is doing has been added to the discussion (page 21, lines 470 - 474).

We have corrected the typos in line 475 and 485.

**Comment:** Thank you for the opportunity to review this important work. I'd be happy to review a revised draft if helpful.
